# Spinal motor evoked responses elicited by transcutaneous spinal cord stimulation in chronic stroke: Correlation between spinal cord excitability, demographic characteristics, and functional outcomes

**DOI:** 10.1371/journal.pone.0312183

**Published:** 2024-11-21

**Authors:** Nicole C. Veit, Chen Yang, Shreya Aalla, Ameen Kishta, Kelly McKenzie, Elliot J. Roth, Arun Jayaraman

**Affiliations:** 1 Department of Biomedical Engineering, Northwestern University, Evanston, Illinois, United States of America; 2 Shirley Ryan AbilityLab, Chicago, Illinois, United States of America; 3 Department of Physical Medicine and Rehabilitation, Northwestern University, Chicago, Illinois, United States of America; Aalto University School of Science and Technology: Aalto-yliopisto Insinooritieteiden korkeakoulu, FINLAND

## Abstract

Transcutaneous spinal cord stimulation (tSCS) is becoming a promising neuromodulation technique to promote motor recovery in various neurological conditions, including stroke. As this intervention moves forward into clinical practice, it is important to understand how the elicited neurophysiological measures are related to the functional and neuromuscular deficits of the population of interest in order to personalize tSCS interventions and assess its effectiveness. Specifically, neurophysiological measurements of spinal cord excitability can be achieved by recording with EMG spinal motor evoked responses (sMERs) in muscles after applying single pulses of tSCS to the spinal cord. The objective of this study was to investigate potential correlations between baseline spinal cord excitability, as measured by resting motor threshold (RMT) and peak-to-peak (P2P) amplitude of the sMERs, and various factors including demographic characteristics, severity of spasticity, muscle strength, and gait speed in individuals post-stroke. Additionally, the study sought to explore disparities in excitability between the paretic and non-paretic sides. Fifteen participants with chronic stroke underwent sMER assessments. We observed a strong positive correlation between RMT and body weight, indicating weight as a potential confounding variable when comparing RMTs from sMERs between individuals. Furthermore, paretic muscles exhibited lower RMTs and higher P2P amplitudes compared to non-paretic muscles. The results demonstrate that sMERs hold promise in uncovering disparities in spinal excitability in stroke participants. Furthermore, careful interpretation and analysis of sMERs is advised, particularly as higher RMTs were associated with higher body weight and could impact the clinical feasibility of tSCS for some participants. These results should be considered in future tSCS protocols that aim to develop more personalized interventions across different neurological populations and optimize sMERs’ utility as an outcome measure.

## Introduction

Transcutaneous spinal cord stimulation (tSCS) is a non-invasive therapeutic modality used predominantly in individuals with spinal cord injury (SCI) to target motor deficits through electrical modulation of spinal networks. Recently, there has been an effort to evaluate the efficacy of this technology in other populations presenting with motor deficits such as multiple sclerosis, Parkinson’s, cerebral palsy, and stroke [1–8]. Specifically for post-stroke lower limb rehabilitation, a few studies have reported positive outcomes when gait training and tSCS are combined [7–9]. However, these studies are predominantly exploratory and present significant variability. Therefore, it is important to understand the meaning of neurophysiological measures evoked by tSCS in the pathophysiology of interest to refine rehabilitation strategies and elucidate tSCS’s mechanism of action. Hence, the objective of this study is to understand neurophysiological responses elicited by tSCS and their relation to the clinical profile of individuals with chronic stroke.

To understand the effects of tSCS in the spinal networks, we perform a neurophysiological assessment to evoke motor responses in the muscles, referred to as spinal motor evoked responses (sMERs). Also known as transpinal evoked potentials (TEPs) [10], posterior root-muscle reflexes (PRMs) [11, 12], or root-evoked potentials (REPs) [13]. sMERs are the compound muscle action potentials measured by surface EMG, elicited by applying single pulses of transcutaneous stimulation to a specific spinal segment. The stimulation is thought to recruit large diameter afferents within the posterior spinal roots, which in turn activate motoneurons and elicit a muscle contraction through the spinal reflex pathway [12, 14]. At higher stimulation intensities, motoneurons could directly be activated through stimulation reaching the anterior roots, although it is less common according to the results presented by Gordineer *et al*. [15, 16]. Resting motor threshold (RMTs) and the peak-to-peak (P2P) amplitude of the sMERs are two metrics that could be obtained from this assessment and have been utilized in previous studies to characterize spinal excitability [6, 10, 11, 17]. Additionally, the RMT obtained from the sMERs assessment is used as a reference for the stimulation threshold intensity to be used during the intervention, as ideally, stimulation intensities should be subthreshold. However, it is unclear whether the sMERs assessment is sensitive enough to 1) reflect the inherent gait deficits of individuals post-stroke, and 2) be used as a physiological outcome measure to assess the effectiveness of a rehabilitation intervention.

For context, it is important to note that other neurophysiological methods such as transcranial magnetic stimulation’s (TMS) motor evoked potentials (MEPs) and H-reflex testing have been employed in stroke rehabilitation research to evaluate neural status [18]. TMS MEPs measure the excitability of the corticospinal tract and have been used as a prognostic metric to predict the recovery of individuals with stroke [19–22]. H-reflexes are the electrical analog of the stretch reflex, which play a crucial role in regulating muscle activity during the different phases of the gait cycle [23]. H-reflex stimulation targets the monosynaptic sensory pathway with electrical stimulation that occurs in the peripheral nerves and individuals post-stroke tend to have higher H-reflex amplitude on their paretic side compared to their non-paretic side [24–28]. However, in the lower limb, the methods studying H-reflex are limited to the tibial nerve, which limits this approach to test and modulate the excitability of a single reflex pathway. In contrast, sMERs may reflect bilateral reflex responses of multiple muscles and therefore could extend the information gained from H-reflex studies [14]. Hence, the sMERs assessment could be an additional neurophysiological tool to understand stroke pathology and dictate stimulation protocols for this population. We explored sMERs’ features and their association to baseline clinical measures as a first step to further understand the usability of this assessment.

In this study, we hypothesized that the characteristics of motoneuron recruitment revealed by sMERs may correspond to the neurophysiological and functional status of individuals with chronic stroke. Our previous work indicated higher motor thresholds in individuals with stroke over healthy controls in sMERs evoked in lower limb muscles [6]. In our current study, we aim to explore the relationship of the clinical profile of individuals with stroke with their spinal cord excitability assessed with tSCS. To do this, we correlated sMERs’ neurophysiological measures, participant demographic characteristics, and clinical scores (spasticity, muscle strength, and gait speed), to identify whether there are any relationships among them. We also explored differences in sMERs between the paretic and non-paretic muscles. Furthermore, we want to inform the field about some implications of the sMERs assessments and considerations when analyzing the data.

## Materials and methods

### Participants

Fifteen participants with stroke (age 58 ± 8 years, 4 ± 2 years post-stroke, 2 females and 13 males) were enrolled in the study from February 13, 2023 to February 26, 2024 (Table 1). The following demographic information was collected: age, chronicity (years since stroke), sex, weight, height, and body mass index (BMI). All participants provided written informed consent. Northwestern University Institutional Review Board (IRB) approved this study (IRB protocol #00215009) and the protocol was in accordance with the criteria set by the Declaration of Helsinki. Participants were recruited based on the following criteria: aged 18 years or older, at least 1-year post-stroke, hemiplegia from a single stroke, not undergoing current physical therapy, and approved by a physician to participate in the study. Exclusions applied to those with ataxia, recent botulinum toxin injections in the lower extremity, Modified Ashworth >3 in the lower extremity, pregnancy or nursing, recent spinal surgery, musculoskeletal dysfunction from injuries or infections, severe lower extremity contractures, active cancer or recent remission, other neurological conditions impacting the study, and unstable cardiorespiratory or metabolic diseases.

**Table 1:** Participants’ demographics.

ID	Years post- stroke	Age	Sex	Weight (kg)	Height (cm)	SSV (m/s)	Orthotic	Type of stroke	MAS (P)	MAS (NP)	MMT (P)	MMT (NP)
1	5	59	F	83	163	0.77	AFO	I	1	0	2.9	5
2	9	56	M	118	175	0.96	Active ankle	I	0	0	4.5	5
3	9	70	M	77	173	0.27	Hinged AFO	I	0.8	0	1.3	5
4	0	66	M	77	175	0.20	Hinged AFO	H	0	0	2.8	5
5	5	55	M	74	173	0.46	None	I	0.5	0	3	5
6	8	57	M	66	168	0.68	None		0.3	0	4.8	5
7	2	54	M	80	175	1.08	Active ankle	I	1.1	0	3.8	5
8	6	71	M	61	175	0.09	Solid AFO	H	1	0.2	3.2	5
9	7	63	M	123	190	0.79	None	I	0.2	0	5	5
10	6	52	M	116	182	1.10	None	H	0.2	0	4.8	5
11	7	48	M	76	172	0.67	Solid AFO	H	0.8	0	1.8	5
12	1	49	M	105	175	1.56	None	H	0	0	5	5
13	1	64	F	84	160	0.37	None	H	1.3	0	3.6	5
14	2	41	M	79	173	0.80	Solid AFO	H	1.6	0	1.9	5
15	4	65	M	83	170	0.32	None		0.3	0	2.8	5
**Mean (sd)**	**4 (2)**	**58 (8)**		**86 (18)**	**173 (7)**	**0.68 (0.39)**			**0.6 (0.5)**	**0 (0.1)**	**3.4 (1.2)**	**5 (0)**

MAS and MMT scores represent the average scores of the four paretic and non-paretic muscles (RF, HAM, MG, TA). MAS scale: 0 (no spasticity) to 4 (max spasticity). MMT scale: 0 (more weakness) to 5 (less weakness).

SSV: self-selected velocity; MMT: manual muscle testing; MAS: modified Ashworth scale; P: paretic; NP: non-paretic; I: ischemic; H: hemorrhagic; F: female; M: male; HAM: medial biceps femoris; RF: rectus femoris; MG: medial gastrocnemius; TA: tibialis anterior

### Spinal motor evoked responses assessment

**Fig 1:**
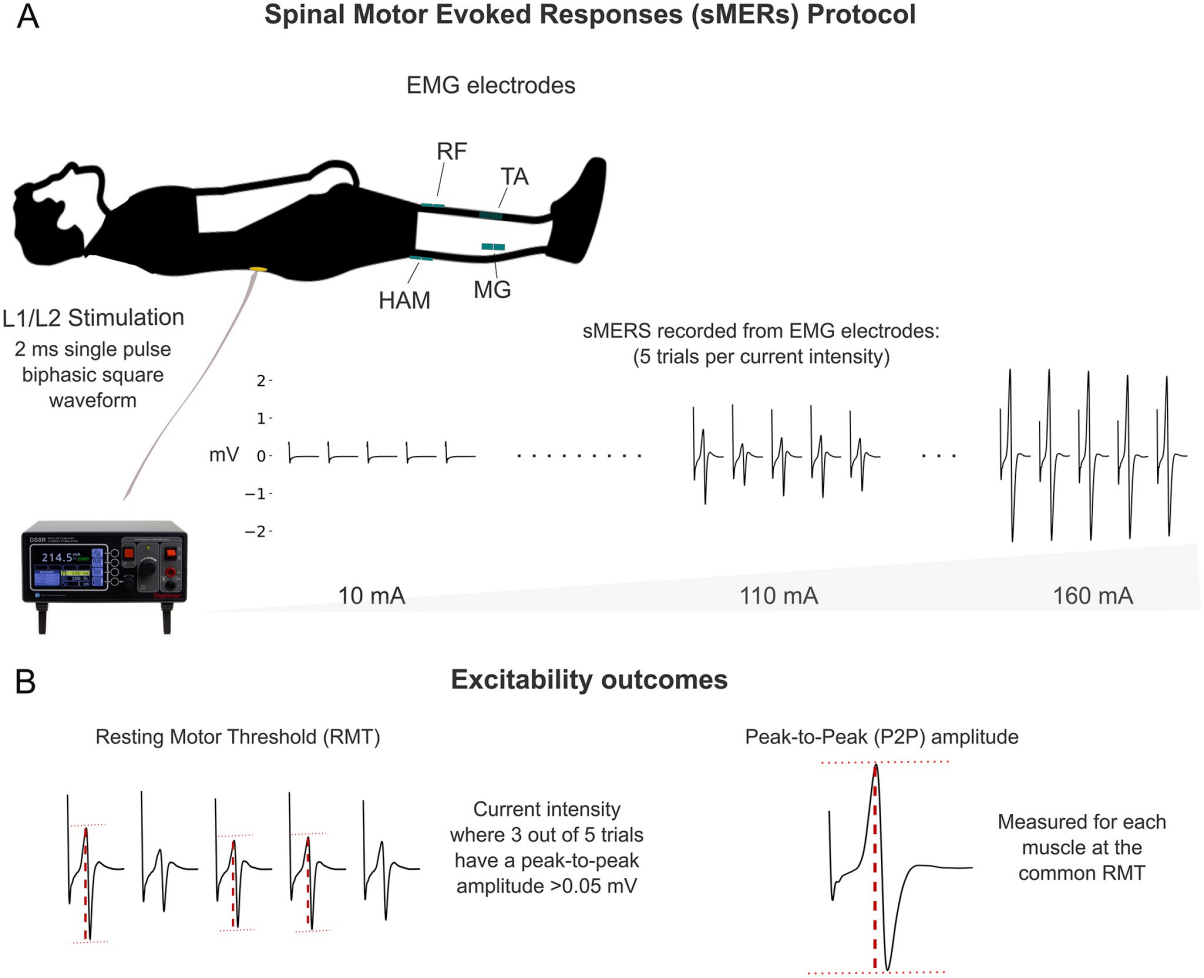
Spinal motor evoked responses (sMERs) protocol setup and neurophysiological measures. A) Sketch of participant laying supine as five single pulses of stimulation are delivered to the L1-L2 spinous processes. EMG data is recorded from HAM, RF, MG, and TA. Representative EMG traces are shown as stimulation intensity increases. B) Definition of the excitability measures obtained from sMERs: resting motor threshold (RMT) and peak-to-peak (P2P) amplitude. HAM: medial biceps femoris; RF: rectus femoris; MG: medial gastrocnemius; TA: tibialis anterior.

Spinal motor evoked responses (sMERs) were performed to assess the muscle activation evoked by a single pulse of tSCS (Fig 1A). A single cathode electrode (3.2 cm diameter, ValuTrode, Axelgaard Ltd., USA) was placed medially at the L1-L2 spinous processes, while a pair of anode electrodes (7.5 X 13 cm, UltraSim, Axelgaard Ltd., USA) were placed symmetrically over the iliac crests. The L1–L2 vertebral levels were selected because they correspond to L2–S2 spinal segments, which is the location of the lumbosacral enlargement of the spinal cord [29]. Participants laid supine as a constant current stimulator (DS8R Digitimer, UK) delivered a single, 2 ms (1 ms per phase), biphasic (cathodic then anodic), symmetric square-wave pulse at 10 mA increments until participant reached maximum tolerance or up to 250 mA. For each stimulation intensity, five pulses were delivered in 5 s intervals.

Surface EMG was used to record the sMERs bilaterally from medial biceps femoris (HAM), rectus femoris (RF), medial gastrocnemius (MG), and tibialis anterior (TA) muscles. Bipolar Ag-AgCl surface electrodes (Easytrode Pregelled Square Electrodes, The Prometheus Group, USA) were placed longitudinally over the belly of the muscles, and reference electrodes were placed bilaterally over the patella. EMG signals were sampled at 4000 Hz using PowerLab 16/35 data acquisition system operated with LabChart software (v7.2, AD Instruments, Bella Vista, NSW, Australia) and filtered (5th order low-pass Butterworth, 450 Hz) using MATLAB (Matworks Inc, USA).

### Clinical outcomes assessment

The Modified Ashworth Scale (MAS, scaled 0 to 4) was used to score the resistance of a relaxed lower limb joint to its full range of motion imposed by a physical therapist [30]. A score of 0 indicates no increase in muscle tone, while higher scores denote progressively increased resistance and therefore the presence of spasticity. The muscles tested included knee flexors (corresponding to HAM in sMERs), knee extensors (RF), ankle dorsiflexors (TA), and ankle plantarflexors (MG). A score of 1+ was replaced with a value of 1.25 during analysis.

The Manual Muscle Testing (MMT) score, ranging from 0 to 5, was used to evaluate the strength of the same muscles evaluated in the Ashworth assessment. A score of 0 signifies no detectable muscle contraction, while a score of 5 indicates normal strength against gravity and resistance. The numbers marked with + and—were modified to add 0.25 or minus 0.25 to the scores, respectively (e.g. 3+ became 3.25).

Table 1 shows the respective MAS and MMT averages for the paretic and non-paretic muscles for each participant.

Three trials of the 10-meter walk test (10MWT) were employed to obtain the average participants’ self-selected velocity (SSV). The test was completed without orthotics or assistive devices, if safe to do so (see Table 1). No physical assistance was provided.

### Data analysis and statistical methods

We assessed two key features for every muscle’s sMER to measure spinal cord excitability: 1) the resting motor threshold (RMT), measured in mA, and 2) the peak-to-peak (P2P) amplitude, measured in mV, of the compound muscle action potential elicited by tSCS (Fig 1B). The RMT for each muscle was defined as the first current intensity where three out of five pulses exhibited a peak-to-peak EMG amplitude response greater than 0.05 mV [31]. The P2P amplitude was obtained for each muscle from the average of the 5 trials at common threshold. The common threshold was defined as the current intensity where all eight muscles reached a P2P amplitude of 0.05 mV. This was chosen to enable a consistent comparison of P2P’s amplitudes at a constant current intensity within each participant. Furthermore, the maximum single-pulse current each participant could tolerate during the sMERs assessment was recorded to investigate if certain demographic factors influence the comfort to stimulation since tolerance to stimulation is a major limitation in tSCS protocols.

To determine whether these metrics were correlated to demographic characteristics, Spearman’s correlations were conducted using the common RMT, the average P2P amplitudes of all muscles at the common RMT intensity, and the maximum tolerance intensity for each subject and correlated it against BMI, weight, height, age, chronicity, and type of stroke. Spearman’s correlation was chosen to capture both monotonic and linear relationships and its robustness to outliers. Given the limited sample size, we employed a permutation test with 1000 permutations to assess the statistical significance (α < 0.05) and confidence intervals of the observed correlation. All comparisons included n = 15 data points, except for type of stroke, where n = 13 due to the lack of this information for two participants.

The relationship of excitability measures (RMT and P2P) to clinical outcomes was explored with partial Spearman’s correlation to account for potential covariate effects. Covariates were derived based on correlations with demographic factors, defining confounders as variables with correlations exceeding 0.60. The three clinical outcomes explored were the following: spasticity (measured by MAS), strength (measured by MMT), and gait speed (SSV). The MAS score and the MMT score of each muscle were correlated to the corresponding muscle’s RMT and P2P, thus a contribution of 8 pairs per participant. Velocity was correlated with the common RMT and average P2P of all muscles at common RMT intensity for each subject. Our analyses aimed to elucidate the impact of spasticity, strength, and gait speed on RMT and P2P, providing insights into how commonly used clinical outcomes might be related to the spinal excitability of individuals with stroke.

To examine neurophysiological differences between the affected (paretic) and unaffected (non-paretic sides) the RMTs and P2P at common RMTs were paired for each muscle and each subject (e.g. paretic HAM with non-paretic HAM). A two-tailed paired t-test was employed for the cases where the differences between pairs were normally distributed, as tested with the Shapiro-Wilk test. Otherwise, a Wilcoxon signed-rank test was used for that muscle pair. This analysis aimed to discern potential asymmetries in the excitability and motor response of the muscles on the paretic and non-paretic sides. To facilitate inter-participant comparisons and report normalized results, the ratio of the P (paretic) side to the NP (non-paretic) side was computed for both RMT and P2P, providing insights into relative muscle activation between the affected and unaffected sides.

All analyses were performed with Python 3.9.15 and statistical significance level was set at 0.05 for all tests.

## Results

### sMERs and demographic characteristics correlation

**Fig 2:**
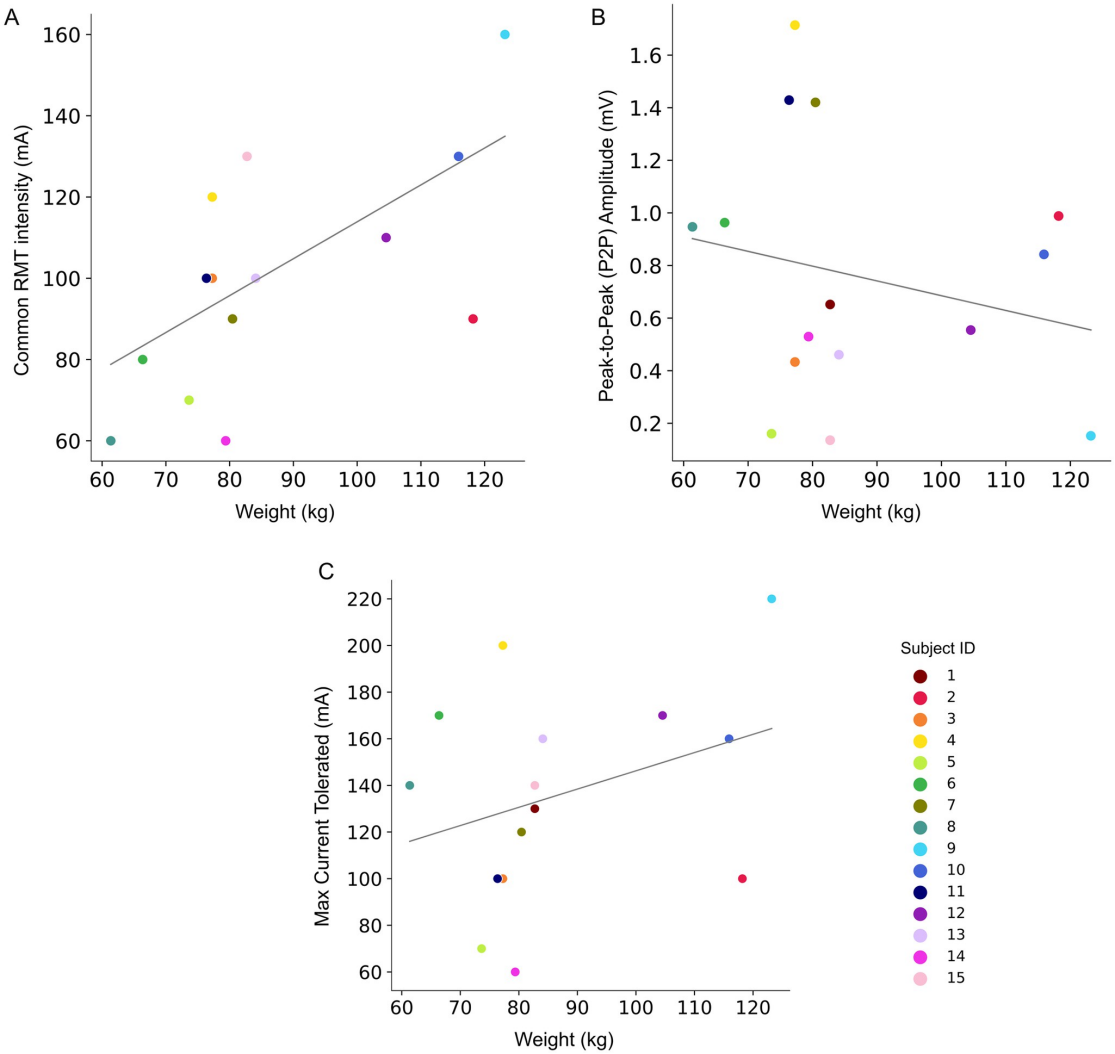
Relationship of weight to sMERs’ outcomes. Weight (kg) plotted against (A) common resting motor threshold (RMT) of all muscles (B) average P2P amplitude of all muscles at common RMT, and (C) maximum current tolerance.

Our analysis revealed that the average common RMTs for all participants was 102.1 ± 27.6 mA. A moderate, positive correlation between common RMT with weight (Spearman’s ρ = 0.63, *p* = 0.014) was observed (Fig 2). No other demographic variables exhibited significant correlations with RMT (Table 2). The P2P average at common RMT intensity was 0.77 ± 1.10 mV and it was not correlated with any of the demographic variables tested. Similarly, the maximum single pulse tolerance intensity was not correlated with any demographic variable and was on average 139.8 ± 43.5 mA.

**Table 2:** Correlations of sMERs outcomes to demographics characteristics.

		Spearman’s coefficient (ρ)	95% CI of ρ	*p* value
Common resting motor threshold (RMT)	BMI	0.56	(0.07, 0.83)	**0.030**
	Weight (kg)	0.63	(0.18, 0.87)	**0.014**
	Height (cm)	0.17	(-0.38, 0.63)	0.536
	Age	0.16	(-0.38, 0.62)	0.574
	Chronicity	-0.09	(-0.57, 0.44)	0.731
	Type of stroke	-0.06	(-0.59, 0.50)	0.846
P2P amplitude at common RMT	BMI	-0.24	(-0.67, 0.31)	0.396
	Weight (kg)	-0.26	(0.68, 0.29)	0.345
	Height (cm)	0.12	(-0.42, 0.59)	0.689
	Age	-0.19	(-0.64, 0.35)	0.485
	Chronicity	-0.01	(-0.52, 0.51)	0.996
	Type of stroke	0.17	(-0.42, 0.66)	0.643
Maximum tolerance intensity	BMI	0.16	(-0.39, 0.62)	0.576
	Weight (kg)	0.29	(-0.26, 0.70)	0.286
	Height (cm)	0.25	(-0.30, 0.67)	0.364
	Age	0.34	(-0.21, 0.73)	0.219
	Chronicity	-0.22	(-0.66, 0.33)	0.418
	Type of stroke	0.13	(-0.46, 0.63)	0.677

### sMERs and clinical scores correlation

Including weight as a covariate to account for its influence on RMT, we explored partial correlations of individual muscle’s RMTs with MAS and MMT clinical scores. A negative, weak trend was observed between RMT and Ashworth score (ρ = -0.17, 95% CI: -0.35 to 0.02, *p* = 0.082, n = 108), suggesting a potential relationship between spinal cord excitability and muscle tone. Correlations between RMT and muscle strength (ρ = 0.09, 95% CI: -0.10 to 0.27, *p* = 0.352, n = 109), as well as gait speed (ρ = -0.44, 95% CI: -0.79 to 0.11, *p* = 0.112, n = 15), were not statistically significant. Additionally, the P2P at the common RMT was weakly correlated to Ashworth (ρ = 0.18, 95% CI: -0.01 to 0.36, *p* = 0.058, n = 107), strength (ρ = -0.22, 95% CI: -0.39 to 0.03, *p* = 0.021, n = 108), and speed (ρ = -0.10, 95% CI: -0.44 to 0.58, *p* = 0.732, n = 15).

### RMTs and P2Ps differences between paretic and non-paretic side

**Fig 3:**
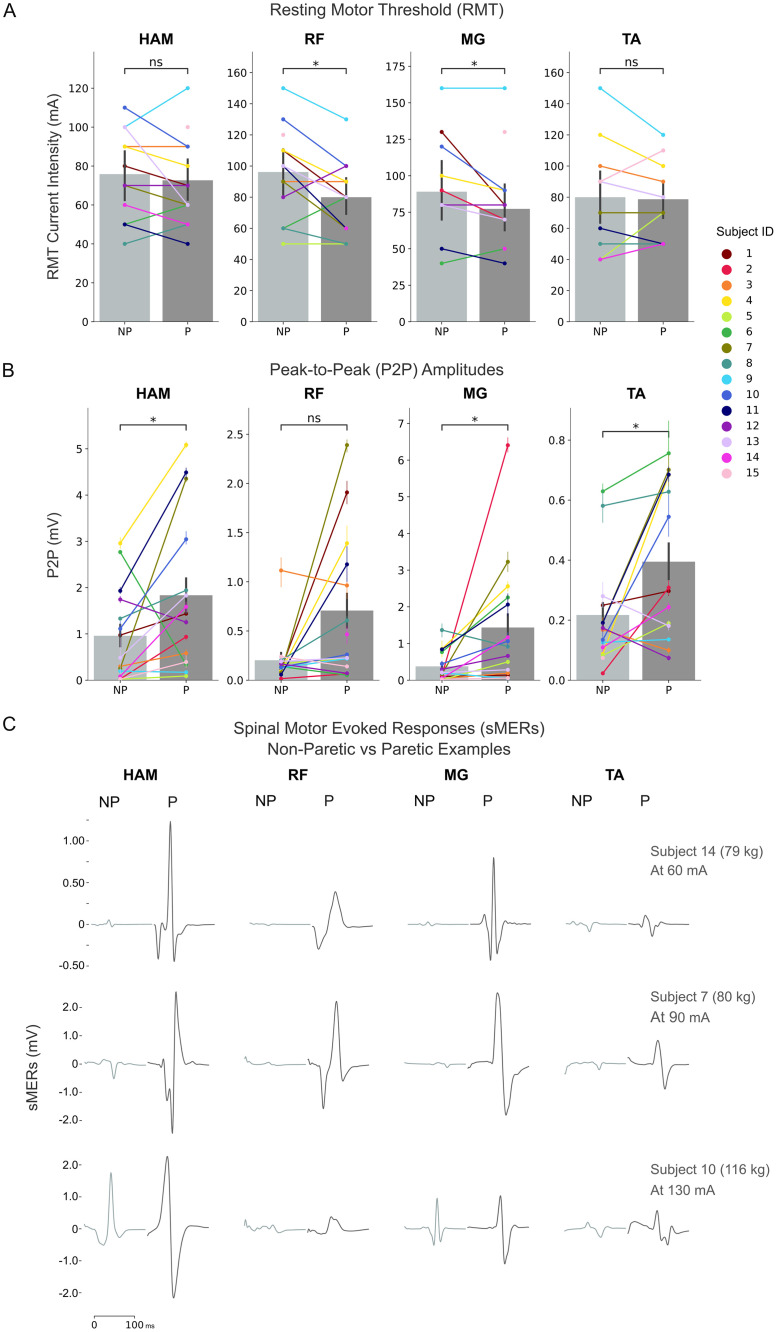
Comparison of sMERs excitability measures between paretic and non-paretic sides. (A) RMT differences between paretic and non-paretic sides for all 4 muscles. (B) P2P at common RMT differences between paretic and non-paretic sides for all 4 muscles. (C) Examples of the sMERs of 3 subjects elicited at common RMT (the current is noted in the right hand corner). HAM: medial biceps femoris; RF: rectus femoris; MG: medial gastrocnemius; TA: tibialis anterior; P: paretic; NP: non-paretic.

We found that RMTs were generally lower on the paretic side compared to the non-paretic side. The differences were only significant for RF (P-NP difference: -13.3 mA, 95% CI: -25.8 to -0.8, *p* = 0.039, Cohen’s d = 0.50) and MG (P-NP difference: -12.7 mA, 95% CI: -23.6 to -1.9, *p* = 0.026, Cohen’s d = 0.36) but not for HAM and TA (Fig 3A, Table 3). Five participants had lower RMTs in the paretic side for all four pairs of muscles (S1, S4, S10, S11, S13). Only one participant exhibited lower RMTs in the non-paretic side for all four pairs of muscles (S6).

**Table 3:** Paired t-test results between the RMTs of paretic and non-paretic muscles.

	Paretic (sd)	Non-Paretic (sd)	Mean of RMT differences (mA)* (95% CI)	Test statistic (N = number of pairs included in test)	*p* value	Cohen’s d	P/NP ratio mean (sd)
HAM	72.7 ± 21.6	75.8 ± 22.7	-5.8 (-15.8, 4.1)	-1.292 N = 12	0.223	0.26	0.94 (0.19)
RF	80.0 ± 22.5	96.2 ± 29.0	-13.3 (-25.8, -0.8)	-2.345 N = 12	* **0.039**	0.50	0.89 (0.22)
MG	77.3 ± 33.1	89.1 ± 36.7	-12.7 (-23.6, -1.9)	-2.609 N = 11	* **0.026**	0.36	0.88 (0.17)
TA	78.7 ± 23.6	80.0 ± 32.3	-0.7 (-10.5, 9.0)	-0.159 N = 14	0.876	0.02	1.05 (0.26)

*Mean difference: paretic side minus non-paretic side.

P: paretic; NP: non-paretic; HAM: medial biceps femoris; RF: rectus femoris; MG: medial gastrocnemius; TA: tibialis anterior; sd: standard deviation

P2P amplitudes at common RMT were higher in the paretic muscles (Fig 3B and 3C). Significant differences indicating greater P2P amplitude in the paretic side compared to non-paretic were noted in all muscles except RF (Table 4). Seven out of 15 participants had higher P2P in the paretic compared to the non-paretic in all four pairs of muscles (S1, S2, S4, S5, S7, S10, S11). None of the participants had higher P2P in the non-paretic compared to the paretic side in all four muscle pairs. Refer to S1 File for detailed recruitment curves displaying the P2P amplitudes of sMERs at all stimulus intensities for all subjects.

**Table 4:** Paired t-test results between the P2Ps at common RMT intensity of the paretic and non-paretic muscles.

	Paretic (sd)	Non-Paretic (sd)	Mean difference of P2P (mV) at common RMT* (95% CI)	Test statistic (N = number of pairs included in test)	*p* value	Cohen’s d	P/NP ratio mean (sd)
HAM	1.84 ± 1.66	0.95 ± 1.00	0.89 (0.06,1.72)	2.288 N = 15	* **0.038**	0.65	7.40 (10.50)
RF‡	0.68 ± 0.73	0.20 ± 0.27	0.49 (0.03, 0.95)	26.0 N = 14	0.104	0.86	7.09 (9.76)
MG‡	1.44 ± 1.70	0.37 ± 0.41	1.07 (0.13, 2.01)	12.0 N = 15	* **0.004**	0.86	10.57 (16.08)
TA	0.39 ± 0.26	0.21 ± 0.18	0.18 (0.04, 0.32)	2.806 N = 14	* **0.015**	0.81	3.10 (3.60)

* Mean difference: paretic side minus non-paretic side.

^‡^ Data was not normally distributed, comparison was made with Wilcoxon signed-rank test.

P: paretic; NP: non-paretic; HAM: medial biceps femoris; RF: rectus femoris; MG: medial gastrocnemius; TA: tibialis anterior; sd: standard deviation

## Discussion

The aim of this study was to explore the relationship between spinal cord excitability, as assessed through motor thresholds (RMT) and amplitudes of the muscle response (P2P) of sMERs, and the clinical outcomes of individuals post-stroke. As TMS MEPs and H-reflex serve to assess the corticospinal tract integrity and a single reflex arc at the periphery, respectively, sMERs could offer a unique perspective by directly assessing multiple muscle responses elicited by stimulation at the spinal cord level. Additionally, the identified RMTs from the sMER assessments serve as a reference of the stimulation intensity to be used during a tSCS intervention, as ideally, the stimulation is subthreshold. Therefore, knowledge of the connection between sMERs’ features and the functional outcomes of individuals post-stroke could aid in personalizing tSCS therapies and refining hypotheses regarding the mechanisms of tSCS, as well as optimizing the use of sMERs assessment for the identification of responders to tSCS. This study represents an initial step in comprehending the clinical significance of sMERs in individuals post-stroke.

sMERs were able to reveal differences in spinal excitability between four paretic and non-paretic muscle pairs. In the present study, the paretic muscles activated at lower current intensities (lower RMT) and higher amplitudes (higher P2P) compared to the non-paretic muscles. This suggests that motoneurons in the paretic side may be more susceptible or sensitive to the incoming afferent signals triggered by tSCS, as tSCS most likely engages the spinal reflex pathways bilaterally by the recruitment of dorsal root sensory afferents to activate motoneurons [32–34]. The reflex hyperexcitability seen in the paretic side has previously been measured by increased H-reflex amplitudes [24], and has been considered an indicator of spasticity and hypertonia due to the stroke leading to a stage of disinhibition of the motoneurons [25, 33]. Interestingly, our results hinted at a subtle trend where lower RMT and higher P2P might be linked to higher spasticity scores, although this association was weak and requires further investigation. Nonetheless, sMERs may have the potential for characterization of baseline spinal cord excitability that could reveal associations with spasticity of multiple muscles and could be used to understand clinical presentations prior to interventions.

Moreover, the absence of significant strong correlations between RMT and P2P concerning muscle strength and walking speed suggests that sMERs may primarily offer direct insights into local spinal neurophysiology. We did observe a weak, significant negative correlation between strength and P2P. However, we hypothesized this might reflect the underlying weakness present in more spastic muscles, where weaker muscles (lower MMT score) had higher P2Ps. Both speed and strength are more complex behaviors that require the involvement of additional neural pathways and depend on inherent muscle properties and adaptations post-stroke that may not be directly reflected in baseline sMERs. For example, the force of an isometric muscle contraction, as measured by MMT, requires a voluntary command that engages the cortex and corticospinal tract. Accordingly, TMS MEPs have been shown to be a better predictor of strength, for which the presence of MEPs in TA was related to greater maximum voluntary contraction, but not gait speed [35]. Gait speed is a more complex metric driven by various corticospinal commands, biomechanical strategies, and motor units firing- timing patterns which may be challenging to be reflected in the measurements of corticospinal or spinal excitability alone [36].

Furthermore, interpretations of RMTs require caution since weight may be a confounding variable. The extent to which body composition versus underlying neurological condition affects RMT is challenging to evaluate without a control group; thus, future studies should include one to better understand this relationship. Our analysis indicated that the weight component of BMI exerts a stronger influence on RMTs compared to height. Increased weight could correspond to more adipose tissue under the stimulation electrodes, leading to higher bioelectrical impedance and explaining this observation. Interestingly, maximum tolerance intensity in the sMERs test was not correlated to body composition, suggesting that tolerance to stimulation may be driven by superficial pain fiber activation and individual subjectivity. Nonetheless, the impact of weight on RMTs should be considered since it might impact the feasibility of tSCS protocols. While P2P amplitudes showed no correlation with weight, interpretations of P2P require careful consideration when comparing absolute values among participants due to various factors affecting EMG output. In this study, we reduced that bias by comparing P2P and RMT absolute values by paired t-tests and ratios between muscle pairs.

Additionally, future studies should explore the sensitivity of sMERs for pre/post intervention measures as these could provide insights into the mechanisms of tSCS. tSCS theories claim to both increase the excitability of local spinal networks to facilitate the reduced corticospinal drive [37] and attenuate the hyperexcitability underlying spasticity [10, 38]. Previous studies have shown lower Ashworth scores in cerebral palsy after tSCS intervention [39–41], and reduction of H-reflex in SCI [10, 42, 43]. Including sMERs as an assessment could reveal if they are sensitive enough to measure changes in excitability and reveal how the intervention is affecting the spinal cord networks.

One limitation of this study was the small sample size, as well as the small range of spasticity scores, and the lack of a control group. Additionally, the protocol was tested only on fixed stimulation parameters (L1-L2 site, biphasic waveform, etc.) and more research needs to be done with other parameters and other clinical populations to validate the relevance of the results. Furthermore, the precise mechanism of SCS recruitment remains unclear. It is highly likely that stimulation activates dorsal root afferents, which, in turn, activate motoneurons monosynaptically through the reflex pathway, though polysynaptic activation is also possible [16]. However, a limitation of this study is that we did not perform a paired stimulus paradigm to confirm whether the elicited motor response was a reflex. In this approach, two stimulation pulses are delivered 25–200 ms apart, and if the P2P amplitude of the second response is reduced compared to the first, it can be labeled as a reflex. This reduction is typically explained by the depletion of neurotransmitter of the recently activated afferent, a phenomenon known as post-activation depression [13]. We recommend the inclusion of this paradigm in studies including sMERs, as it could provide valuable insights into the mechanisms of tSCS. Along with that point, the precise neural activation achieved by placing the electrode on the L1-L2 vertebrae is not fully understood. It is known that the spinal cord termination varies from T12 to the L3 vertebra [44]. Therefore, the specific spinal segment and dorsal afferent directly beneath the L1-L2 vertebral electrode location may vary between individuals, contributing to intersubject variability in muscle recruitment. In adults, the cauda equina is likely to be present at the L1-L2 vertebrae, therefore stimulating at that site may lead to the recruitment of a broader range of dorsal roots, including those associated with the lumbosacral enlargement spinal segments. This could explain why we still observed activation of lower limb muscles in all participants. Despite potential variability among electrode positioning across subjects, we consistently observed differences in excitability between paretic and non-paretic sides and future studies should further understand how varying electrode position might affect this finding. Lastly, more objective clinical assessment methods should be used in the future, including measurements of the skin fat with a caliper at the spinal electrode site and the measurements of spasticity and strength.

## Conclusions

In summary, this study associated neurophysiological baseline measurements elicited by tSCS with post-stroke baseline demographic characteristics and clinical outcomes. We revealed differences in sMERs’ paretic and non-paretic sides that may be related to hyperreflexive behaviors such as spasticity. These findings suggest that desired neurophysiological outcome after a tSCS intervention in stroke may involve decreasing spinal cord excitability in the paretic side, unlike in pathologies such as SCI where enhancement is sought. Additionally, it raises the question of whether electrode placement in tSCS interventions for stroke should be adjusted, possibly towards lateral positioning, to selectively target one side over the other. Another significant finding concerns the influence of weight on the motor thresholds. While the resting motor threshold has been indicative of the functional engagement of sensorimotor networks, our study highlights the impact of body composition on this measure. This underscores the importance of caution when comparing values between participants. Furthermore, this finding holds implications for identifying individuals for whom tSCS intervention might be feasible and tolerate stimulation, as stimulation should be delivered close to their motor thresholds and tolerance is not affected by weight of the participant. We hope that the results of this study can provide guidance for creation of personalized tSCS interventions as well as optimizing sMERs’ utility as an outcome measure. Since the precise mechanism of tSCS for stroke rehabilitation remains elusive, forthcoming studies in tSCS and stroke should include sMERs as an assessment and report pre/post changes in RMTs and P2P as well as the asymmetries noticed between paretic and non-paretic muscles. This may provide insight as to the underlying mechanisms of stroke recovery and pave the way for innovation in rehabilitation assessments and interventions utilizing tSCS.

## Supporting information

S1 File Recruitment curves for all muscles and subject. Recruitment curves showing the average peak-to-peak (P2P) amplitude of sMERs at each stimulus intensity for all muscles (HAM, RF, MG, TA) in 15 subjects. Each muscle’s data is divided into paretic (P) and non-paretic (NP) sides.
(PDF)

S2 File Excel file containing current intensity and P2P data at RMT, common RMT, and maximum current intensity for all muscles and subjects. (XLSX)
