# Supplementary material for: Spinal motor evoked responses elicited by transcutaneous spinal cord stimulation in chronic stroke: Correlation between spinal cord excitability, demographic characteristics, and functional outcomes
Source: PLoS One. 2024 Nov 21;19(11):e0312183. doi: 10.1371/journal.pone.0312183 (PMC11581260; doi:10.1371/journal.pone.0312183)
Supplement: S1 File — Recruitment curves showing the average peak-to-peak (P2P) amplitude of sMERs at each stimulus intensity for all muscles (HAM, RF, MG, TA) in 15 subjects. Each muscle’s data is divided into paretic (P) and non-paretic (NP) sides. (PDF) [file pone.0312183.s001.pdf]

Supplementary File 1. Recruitment curves showing the average peak-to-peak (P2P) amplitude of sMERs at each stimulus intensity for all muscles (HAM, RF, MG, TA) in 15 subjects. Each muscle's data is divided into paretic (P) and non-paretic (NP) sides.

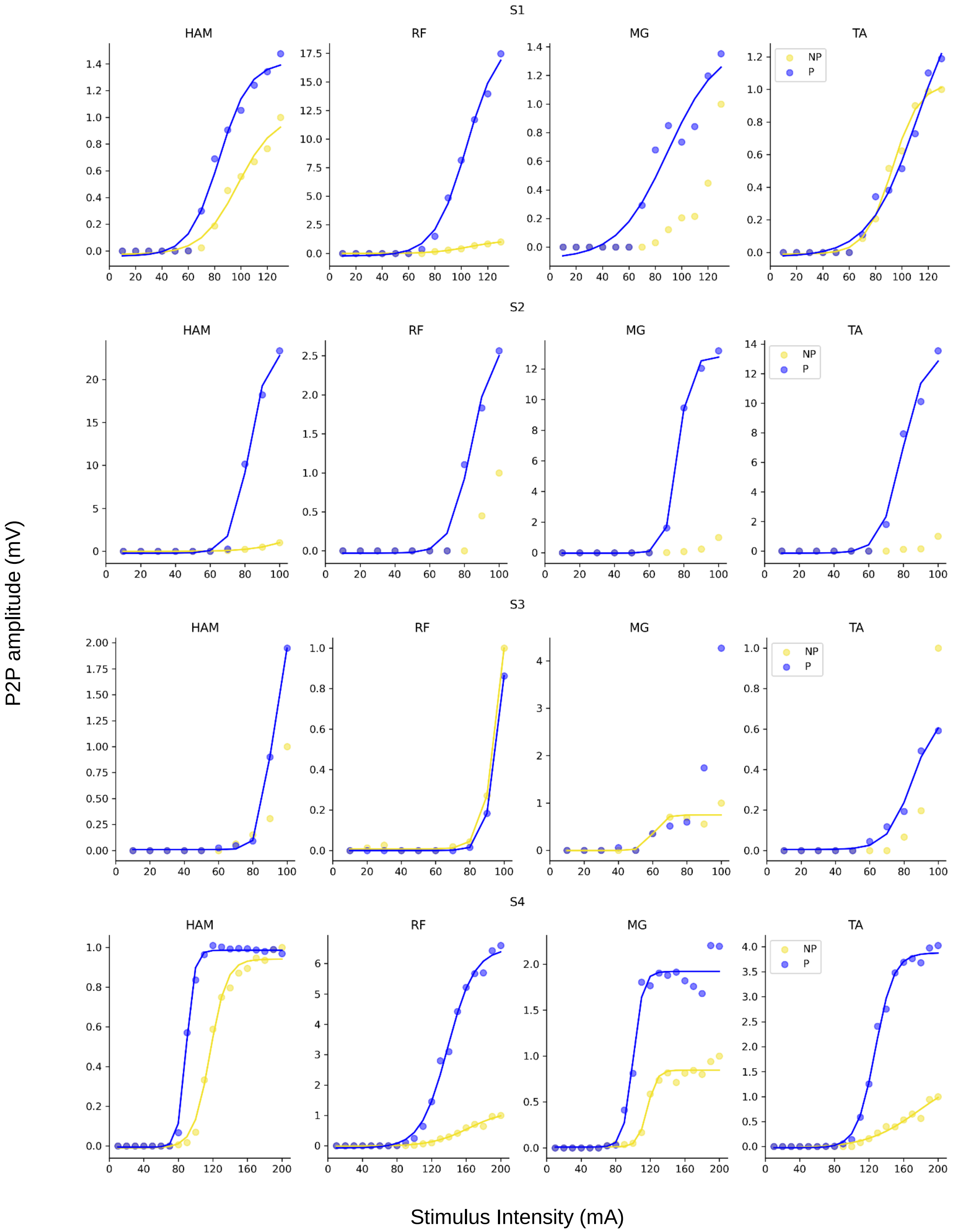

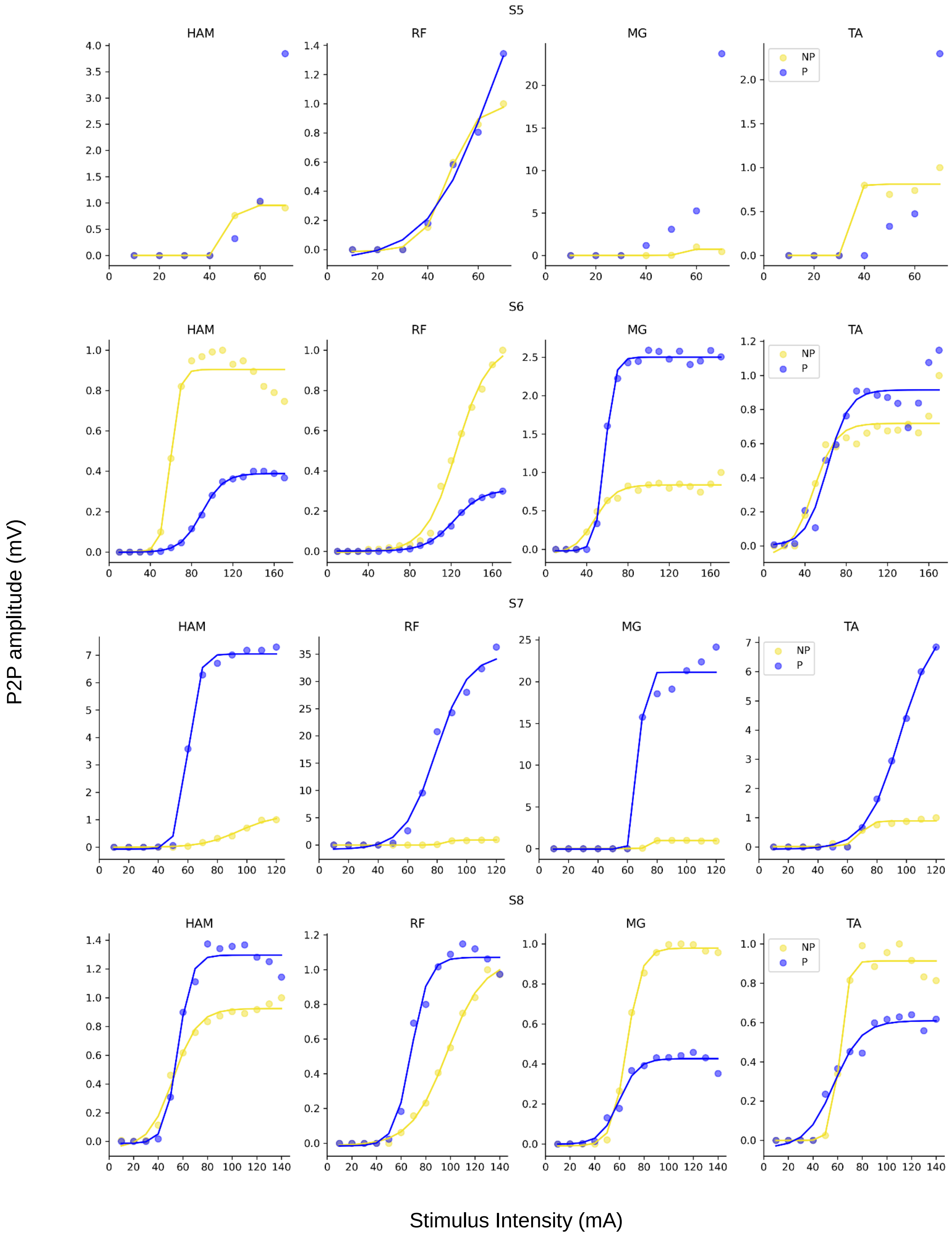

S5
S6
S7
S8
HAM
RF
MG
TA
NP
P
P2P amplitude (mV)
Stimulus Intensity (mA)

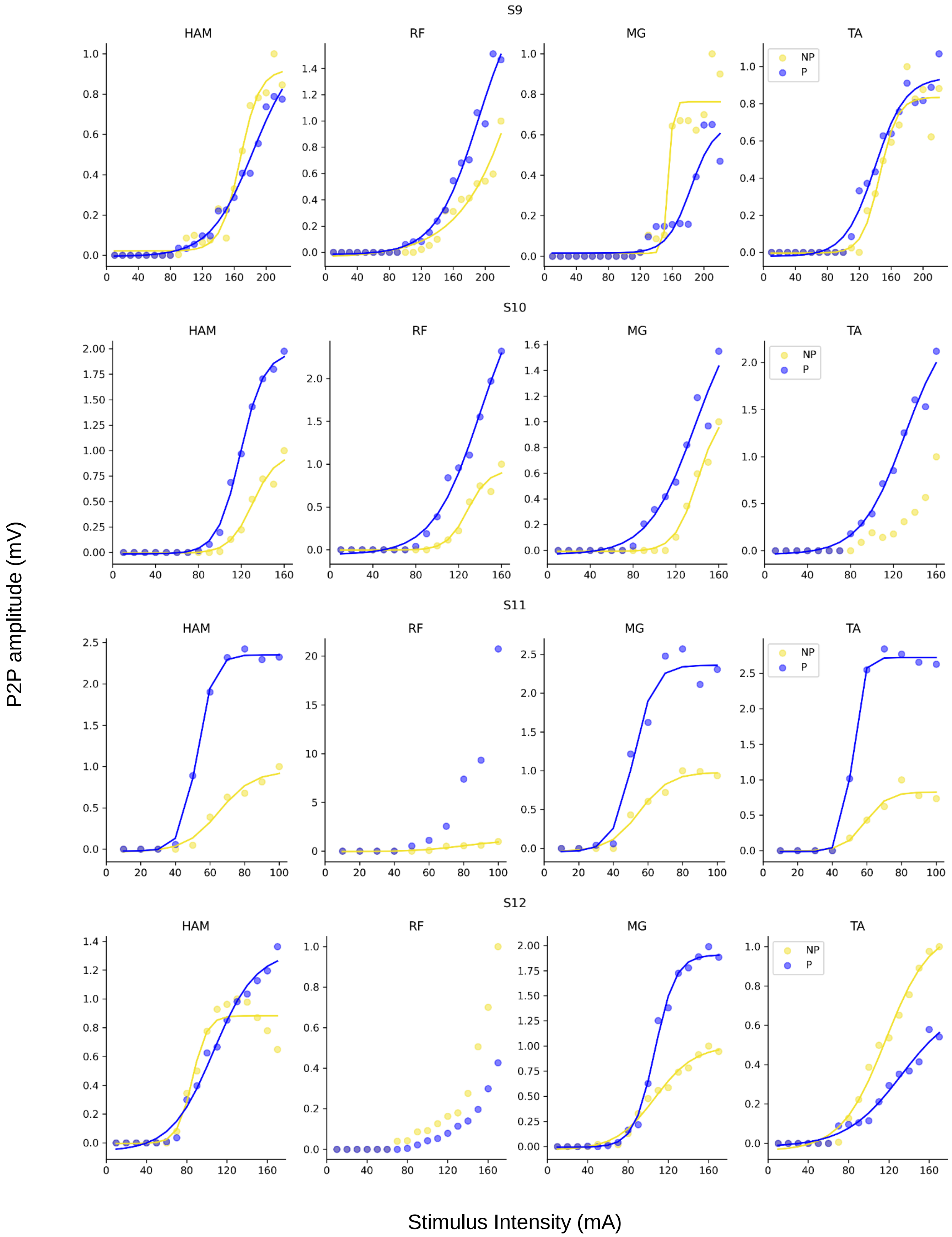

S9
HAM
RF
MG
TA
NP
P
S10
S11
S12
P2P amplitude (mV)
Stimulus Intensity (mA)

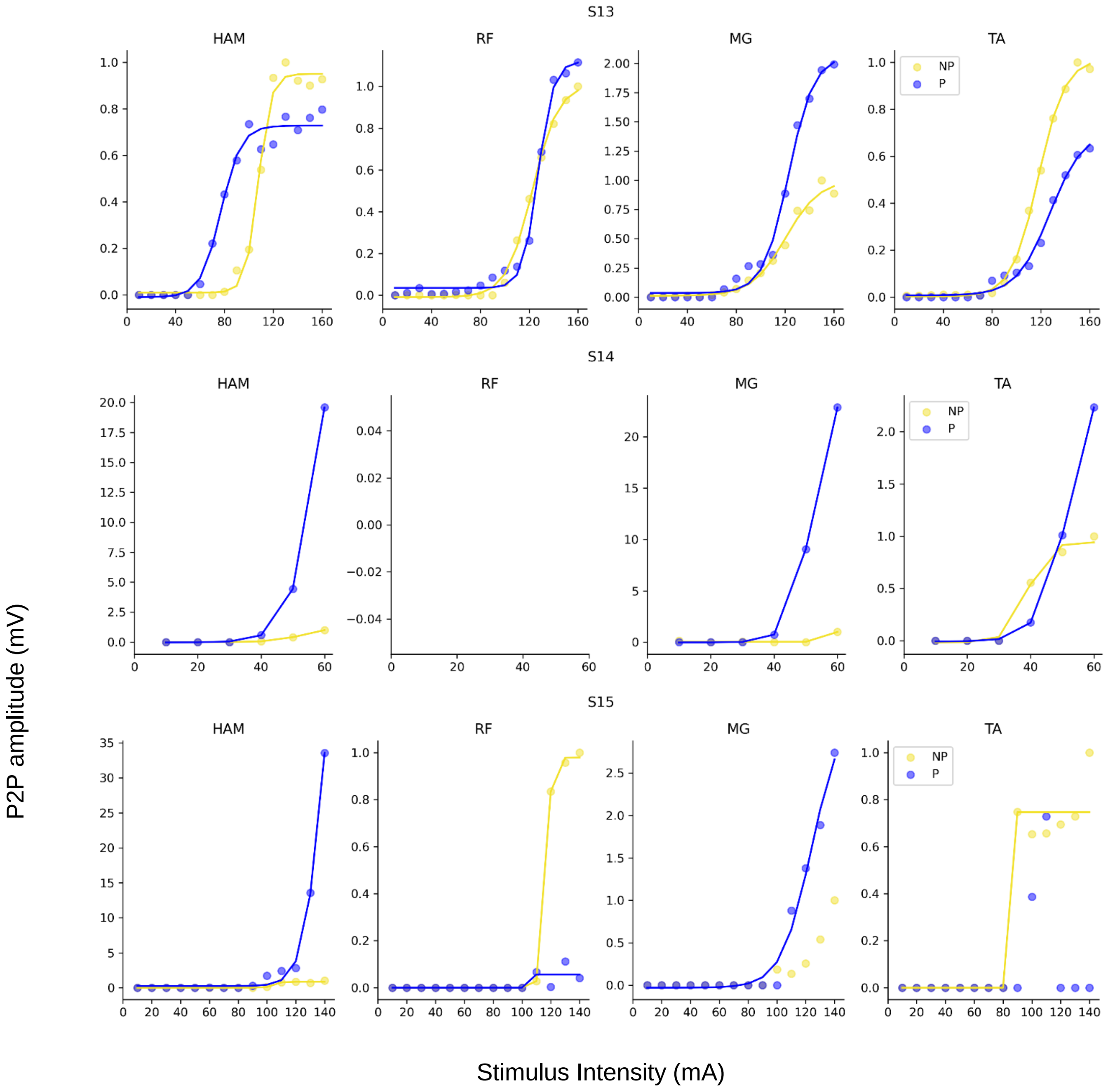
S13
HAM
RF
MG
TA
NP
P
S14
HAM
RF
MG
TA
NP
P
S15
HAM
RF
MG
TA
NP
P
P2P amplitude (mV)
Stimulus Intensity (mA)
